# Editorial: Brain cholinergic mechanisms

**DOI:** 10.3389/fnsyn.2015.00014

**Published:** 2015-09-15

**Authors:** Sukumar Vijayaraghavan, Geeta Sharma

**Affiliations:** Department of Physiology and Biophysics and the Neuroscience Program, School of Medicine, University of ColoradoAurora, CO, USA

**Keywords:** acetylcholine, nicotinic, muscarinic, interneurons, synaptic

While historically acetylcholine (ACh) holds a central place in the discovery of chemical transmission in the nervous system, progress in our knowledge of the mechanistic underpinnings of cholinergic transmission in the central nervous system (CNS) has lagged behind its sister transmitters. For example, unlike what we know about glutamatergic transmission, neither the prevalence of fast cholinergic transmission or postsynaptic specializations at cholinergic synapses, is well understood. Every level of inquiry into the cholinergic system reveals a bewildering complexity in signaling and transduction mechanisms. Receptor localizations within and among neurons, along with transmitter source and access to receptors, provide for a system that is capable of neuromodulation at multiple time scales that allow for short- and long-term regulation of circuit output in the brain. The compilation of reviews and primary papers in this research topic provides a sampling of findings at different levels of integration that highlight both the current status of our understanding of CNS cholinergic mechanisms as well as reveals the gaps in our knowledge that need to be filled.

How ACh mediates signaling in the brain is still an unresolved issue at the level of synaptic physiology. Cholinergic innervation in the brain arises from two main loci- the basal forebrain and the pendunculo-pontine area of the hindbrain. In both instances a relatively small cluster of neurons send diffuse projections into various target areas. The diffuse nature of the projections, as well as the non-planar relationship between the cholinergic neurons and their targets precludes traditional slice electrophysiology approaches to examining signaling. Recent advances in optogenetic techniques offer a potential solution to this problem and some of the advances made thus far are reviewed in this research topic (Luchicchi et al., 2014). Some of the relevant issues that need to be resolved are the prevalence of synaptic vs. non-synaptic modes of activation, the role of transmitter diffusion, relationship between ACh release sites and the distribution of acetylcholine esterases, and the potential co-release of other transmitters from cholinergic terminals.

In addition to the centrifugal cholinergic inputs, areas of the cortex also express their own, resident, cholinergic interneurons. Well studied among these are the cholinergic interneurons of the striatum, relevant to disorders like Parkinson's disease. These are large aspiny neurons that are small in number (about 1% of the total neuronal population) but send dense projections throughout the area. Recent findings that are summarized here (Lim et al., 2014) show that these neurons themselves are subject to complex regulation, both excitatory and inhibitory, from a vast area of other transmitter systems. Another area, where cholinergic interneurons have been described is the hippocampus (Frotscher et al., 2000). Thus, far, these neurons have remained an oddity, with no functional attribution. In this research topic, work done by the group of Josh Lawrence (Yi et al., 2015) has begun the examination of these neurons using transgenic mice where GFP or YFP is expressed driven by the choline acetyltransferase promoter. Interestingly, these neurons might themselves be subject to cholinergic modulation, presumably from basal forebrain innervation.

We have a long way to go before we understand the complex variables of cholinergic signaling. Differential expression of various subtypes of the ionotropic nicotinic acetylcholine receptors (nAChRs) and the metabotropic muscarinic acetylcholine receptors (mAChRs), their expression in multiple neuronal types within a region, and varying locations within a neuron (i.e., somatic, dendritic, synaptic etc.) all orchestrate a complex symphony of neuromodulation. A review of receptor localization and function within hippocampal CA 1 interneurons (McQuiston, 2014) illustrates how complex expression of the two classes of receptors can allow for differential control of principal cell activity under varying concentrations of the transmitter.

These local control mechanisms can regulate circuit outputs in various regions of the brain, potentially mediating the attention and learning-specific behaviors ascribed to ACh-driven modulation. In the olfactory system, nAChR activation can filter odor signals such that weak inputs are eliminated while strong ones are allowed through, thus providing a mechanism for altering the gain function of a circuit (D'souza and Vijayaraghavan, 2014) and, perhaps, regulating the effects of these receptors on odor discrimination (Hellier et al., 2010). In the visual cortex, differential functional expression of mAChRs might explain neuronal synchrony and gamma oscillations allowing for the tuning of network output during tasks involving perceptual learning (Groleau et al., 2015). In the hippocampus, a potential role for mAChR-regulated release of endocannabinoid in circuit oscillation highlight the complex mechanisms in play that underlie the effects of the cholinergic system in regulation of behavior (Alger et al., 2014).

Cholinergic receptors are a potential gold mine as targets for therapies and pharmaceutical companies have not been diffident about exploring various receptor ligands as potential therapies. Emerging studies implicate cholinergic receptors in various addictive mechanisms: the direct interaction between cocaine and nAChRs reported in this research topic (Acevedo-Rodriguez et al., 2014) illustrates how these receptors could play an important role in the reinforcement properties of cocaine. The cholinergic system appears to be involved in specific behavioral endophenotypes of a number of diseases such as Alzheimer's disease (Oddo and LaFerla, 2006), schizophrenia (Wallace and Bertrand, 2013), and Parkinson's disease (Bohnen et al., 2010). More recent animal model studies also implicate the transmitter system in autism (Amodeo et al., 2014).

This is prime time for the development of clinical therapies that target the cholinergic system for a host of brain disorders. At the same time, as the collection of studies in this research topic illustrates, much remains to be understood regarding the physiology of cholinergic transmission and modulation. There is a risk of pharmacological advances outpacing our knowledge of cholinergic signaling mechanisms in the brain. History tells us that such a disconnect between therapeutic advances and our knowledge of physiology can often lead to unintended complications from novel therapies, a classic example being the aggressive marketing of heroin as a cough remedy at the turn of the twentieth century. There needs to be significant investment in examining the basic biology of cholinergic modulation of brain circuits in order to develop more rational and safe therapeutic targets that the cholinergic system offers.

## Conflict of interest statement

The authors declare that the research was conducted in the absence of any commercial or financial relationships that could be construed as a potential conflict of interest.
